# Non-mercaptalbumin, Oxidized Form of Serum Albumin, Significantly Associated with Renal Function and Anemia in Chronic Kidney Disease Patients

**DOI:** 10.1038/s41598-018-35177-x

**Published:** 2018-11-14

**Authors:** Shinya Nakatani, Keiko Yasukawa, Eiji Ishimura, Ayumi Nakatani, Norikazu Toi, Hideki Uedono, Akihiro Tsuda, Shinsuke Yamada, Hitoshi Ikeda, Katsuhito Mori, Masanori Emoto, Yutaka Yatomi, Masaaki Inaba

**Affiliations:** 1 Department of Metabolism, Endocrinology, and Molecular Medicine, Osaka City University Graduate School of Medicine, Osaka, Japan; 2 Department of Clinical Laboratory, The University of Tokyo Hospital, Tokyo, Japan; 3 Department of Nephrology, Osaka City University Graduate School of Medicine, Osaka, Japan

## Abstract

Oxidative stress plays a major role in development of cardiovascular disease in patients with chronic kidney disease (CKD). Human mercaptalbumin (HMA), a reduced form of serum albumin, and non-mercaptalbumin (HNA), an oxidized form of serum albumin, are known as indicators for evaluating oxidative stress in systemic circulation, including end-stage renal disease cases. We investigated factors associated with fraction of HNA [f(HNA)] in 112 pre-dialysis CKD patients (63.6 ± 14.0 years old; 59 males, 53 females) using a newly established anion-exchange column packed with hydrophilic polyvinyl alcohol gel as well as high performance liquid chromatography. Mean f(HNA) in our CKD patients was 30.0 ± 6.1%, higher than that previously reported for healthy subjects. In multiple regression analysis, age (β = 0.200, p = 0.014), eGFR (β = −0.238, p = 0.009), hemoglobin (β = −0.346, p < 0.001), and ferritin (β = 0.200, p = 0.019) were significantly and independently associated with f(HNA) (R^2^ = 0.356, p < 0.001). In addition, factors related to CKD-mineral and bone disorder (CKD-MBD), including intact-PTH (β = 0.218, p = 0.049) and 1,25-dihydroxyvitamin D (1,25(OH)_2_D) (β = −0.178, p = 0.040), were significantly and independently associated with serum f(HNA) (R^2^ = 0.339, p < 0.001), whereas fibroblast growth factor-23 was not. These findings indicate the importance of management of hemoglobin and ferritin levels, as well as appropriate control of CKD-MBD factors for a better redox state of serum albumin in CKD patients.

## Introduction

Chronic kidney disease (CKD) and end-stage renal disease (ESRD) are strongly associated with cardiovascular disease (CVD)^1^. Oxidative stress, which is involved with production of excessive levels of reactive oxygen species (ROS), is closely related to the progression of CKD^2^, while it has also been proposed to play a major role in development of CVD in CKD patients^3^.

Human serum albumin (HSA) is a simple protein comprised of 585 amino acids with a molecular weight 66 kD and produced in the liver^4^. Among the 35 HSA cysteine residues, only N-terminal cysteine 34 (Cys34) remains free. HSA is capable of scavenging hydroxyl radicals with its reduced (-SH) cysteine residue Cys34. Based on the state of Cys34, HSA exists in 2 main forms; the oxidized form of human non-mercaptalbumin (HNA) and reduced form of human mercaptalbumin (HMA)^5,6^.

HNA and HMA have been shown to be good indicators for evaluating oxidative stress in systemic circulation of various types of patients, including those with chronic liver failure^7,8^ and ESRD^9,10^. A higher HNA level has been reported to be a considerable risk factor for CVD in patients undergoing hemodialysis and peritoneal dialysis^10,11^. Thus, it is clinically important to measure HNA and HMA in patients with an elevated risk of CVD, *i*.*e*., those with CKD and ESRD. However, few investigations have examined the relationship between serum fraction of HNA [f(HNA)], a serum marker of oxidative stress determined using the formula HNA/(HNA + HMA)*100^9,12^, and clinical parameters in pre-dialysis CKD patients^13^.

Conventional methods for measurements of HNA and HMA have been developed, including high performance liquid chromatography (HPLC)^14^, HPLC-post column bromocresol green^15^, and anion exchanging chromatography^6^. However, the protocols used for analysis with those are complicated and/or extremely time consuming, and a more rapid and sensitive clinical laboratory method for assessment of HNA and HMA has been anticipated. Therefore, we recently developed a novel method to measure serum HNA and HMA that utilizes an anion-exchange column packed with hydrophilic polyvinyl alcohol gel, along with HPLC^12^. This assay technique features a shorter analytical time, and provides highly accurate and reproducible measurements of HNA and HMA^12^. In healthy adults examined with this system, approximately 20–25% of serum albumin has been shown to be HNA^9,12^, with the remaining 75–80% found to be HMA^16^.

In the present study, we conducted a cross-sectional single center investigation of 112 CKD patients, in whom we measured serum HNA and HMA using our newly developed method. With these results, the relationship between f(HNA) and clinical CKD-related parameters was examined.

## Results

### Clinical characteristics of patients with pre-dialysis CKD

The clinical characteristics of the CKD patients are presented in Table 1. Average (±SD) age, serum creatinine level, and eGFR were 63.6 ± 14.0 years, 2.2 ± 1.4 mg/dL, and 30.7 ± 15.6 mL/min/1.73 m^2^, respectively. Percent f(HNA) was calculated using the formula HNA/(HNA + HMA)*100^9,12^. The mean value in the present patients was 30.0 ± 6.1%, which was higher than that previously reported for healthy subjects [f(HNA) 25.1 ± 3.0%; age 63.1 ± 9.9 years, serum creatinine 0.80 ± 0.19 mg/dL]^9^. Sixty-eight of 112 patients were given renin-angiotensin-aldosterone system (RASS) inhibitors. However, f(HNA) was not significantly different between CKD patients with and without RAAS inhibitor administration (30.4 ± 7.6% *vs*. 30.2 ± 5.9%, p = 0.809).

**Table 1:** Clinical characteristics of 112 CKD patients.

Males/females (no.)	59/53
Age (years)	63.6 ± 14.0
f(HNA) (%)	30.0 ± 6.1
Body mass index (kg/m^2^)	22.5 ± 4.4
eGFR (mL/min/1.73 m^2^)	30.7 ± 15.6
Creatinine (mg/dL)	2.2 ± 1.4
Blood urea nitrogen (mg/dL)	33 ± 20
Aspartate transaminase (IU/L)	20 ± 6.7
Alanine transaminase (IU/L)	16 ± 9.4
Total protein (g/dL)	6.9 ± 0.7
Albumin (g/dL)	3.8 ± 0.5
C-reactive protein (mg/dL)	0.23 ± 0.4
Uric acid (mg/dL)	6.5 ± 1.5
Hemoglobin (g/dL)	12.5 ± 1.6
Ferritin (ng/mL)	105 ± 105
Transferrin saturation (%)	27 ± 11
Plasma glucose (mg/dL)	105 ± 32
Hemoglobin A1c (%)	5.7 ± 0.6
Sodium (mEq/L)	141 ± 2.5
Potassium (mEq/L)	4.5 ± 0.5
Chloride (mEq/L)	107 ± 3.3
Sodium-chloride (mEq/L)	33.7 ± 2.9
Corrected calcium (mg/dL)	9.4 ± 0.7
Phosphate (mg/dL)	3.9 ± 0.8
Intact-PTH (pg/mL)	104 ± 106
Fibroblast growth factor-23 (pg/mL)	116 ± 1106
1,25-dihydroxyvitamin D (pg/mL)	38.4 ± 16.2
Urinary protein (g/gCr)	2.3 ± 1.9
ESA user/non-user	26/86
Iron supplementation, yes/no	18/94
ARB, yes/no	67/45
ACEI, yes/no	1/111
Calcium antagonist, yes/no	56/56
Active vitamin D, yes/no	27/85
Phosphate binders, yes/no	9/103
AST-120, yes/no	9/103

eGFR: estimated glomerular filtration rate, PTH: parathyroid hormone, ESA: erythropoiesis-stimulating agents. ARB: angiotensin II receptor blocker, ACEI: angiotensin-converting enzyme inhibitor. Values are expressed as the mean ± SD, as appropriate.

### Correlations between f(HNA) and clinical parameters in CKD patients

**Figure 1:**
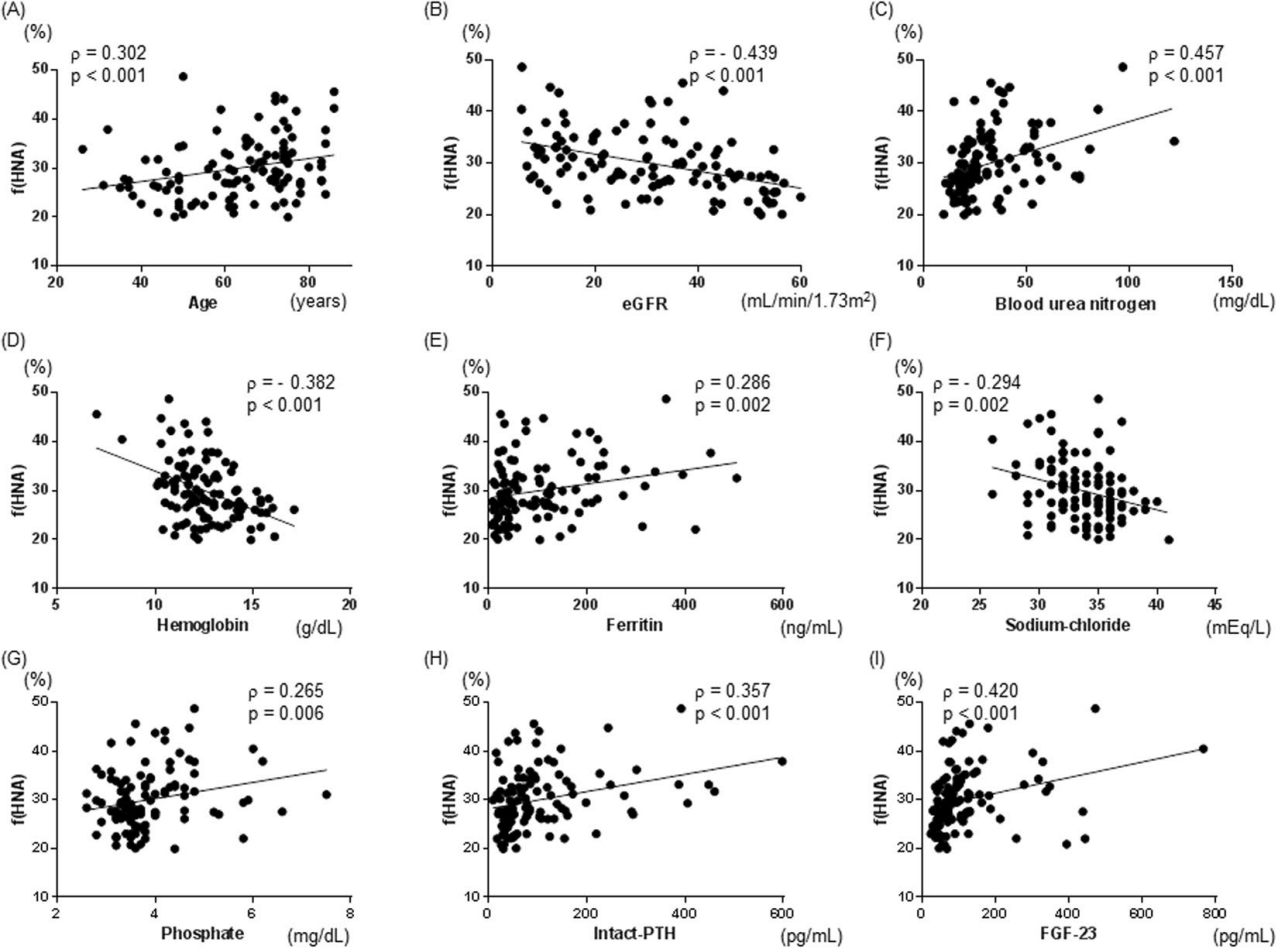
Correlations between serum fraction of human non-mercaptalbumin [f(HNA)] and clinical parameters in CKD patients. Age (**A**), blood urea nitrogen (**C**), ferritin (**E**), phosphate (**G**), intact-PTH (**H**) and FGF-23 (**I**) were significantly and positively correlated with f(HNA). Estimated glomerular filtration rate (eGFR) (**B**), hemoglobin (**D**), and sodium-chloride (**F**) were significantly and negatively correlated with f(HNA).

The correlations between patient clinical parameters and f(HNA) were examined using simple regression analysis (Table 2). Age and levels of creatinine, blood urea nitrogen, ferritin, chloride, phosphate, intact-parathyroid hormone (PTH), and FGF-23 were significantly and positively correlated with f(HNA) in our CKD patients (ρ = 0.302, p < 0.001; ρ = 0.410, p < 0.001; ρ = 0.457, p < 0.001; ρ = 0.286, p = 0.002; ρ = 0.230, p = 0.015; ρ = 0.265, p = 0.006; ρ = 0.357, p < 0.001; ρ = 0.420, p < 0.001, respectively) (Table 2, Fig. 1). Furthermore, eGFR, and levels of hemoglobin and sodium-chloride, the latter a marker of metabolic acidosis^17^, were also significantly and negatively correlated with f(HNA) (ρ  = −0.439, p < 0.001; ρ = −0.382, p < 0.001; ρ = −0.294, p = 0.002, respectively) (Table 2, Fig. 1).

**Table 2:** Correlations between clinical parameters and serum fraction of human non-mercaptalbumin [f(HNA)] in 112 CKD patients (simple regression analysis).

	ρ	p
Age (years)	0.302	<0.001
Body mass index (kgm^2^)	0.084	0.377
eGFR (mL/min/1.73 m^2^)	−0.439	<0.001
Creatinine (mg/dL)	0.410	<0.001
Blood urea nitrogen (mg/dL)	0.457	<0.001
Aspartate transaminase (IU/L)	−0.010	0.294
Alanine transaminase (IU/L)	−0.172	0.074
Total protein (g/dL)	−0.054	0.578
Albumin (g/dL)	−0.170	0.074
C-reactive protein (mg/dL)	0.139	0.145
Uric acid (mg/dL)	0.019	0.840
Hemoglobin (g/dL)	−0.382	<0.001
Ferritin (ng/mL)	0.286	0.002
Transferrin saturation (%)	0.048	0.614
Plasma glucose (mg/dL)	0.144	0.129
Hemoglobin A1c (%)	0.093	0.331
Sodium (mEq/L)	0.029	0.765
Potassium (mEq/L)	0.086	0.365
Chloride (mEq/L)	0.230	0.015
Sodium-chloride (mEq/L)	−0.294	0.002
Corrected calcium (mg/dL)	−0.131	0.170
Phosphate (mg/dL)	0.265	0.006
Intact-PTH (pg/mL)	0.357	<0.001
Fibroblast growth factor-23 (pg/mL)	0.420	<0.001
1,25-dihydroxyvitamin D (pg/mL)	−0.138	0.156
Urinary protein (g/gCr)	0.295	0.002

eGFR: estimated glomerular filtration rate, PTH: parathyroid hormone, g/gCr: gram per gram of creatinine.

### Multivariate analysis of clinical parameters associated with f(HNA)

Next, we investigated clinical parameters associated with f(HNA) using multivariate analysis, with age, gender, body mass index (BMI), aspartate transaminase (AST), hemoglobin (marker of renal anemia), uric acid, sodium-chloride (marker of metabolic acidosis), intact-PTH [marker of CKD mineral and bone disorder (CKD-MBD)], eGFR, and urinary protein included as explanatory variables. The results showed that age (β = 0.255, p = 0.005), hemoglobin (β = −0.341, p = 0.001), intact PTH (β = 0.221, p = 0.039), and eGFR (β = −0.262, p = 0.003) were significantly and independently associated with serum f(HNA) in our CKD patients (R^2^ = 0.365, p < 0.001) (Table 3).

**Table 3:** Multivariate analysis of various clinical CKD factors associated with serum fraction of human non-mercaptalbumin [f(HNA)].

	*β*	*p*
Age	0.255	0.005
Gender	−0.121	0.185
Body mass index	0.132	0.149
Aspartate Transaminase	0.138	0.129
Hemoglobin	−0.341	0.001
Uric acid	−0.054	0.524
Sodium-chloride	0.088	0.417
Intact-PTH	0.221	0.039
eGFR	−0.262	0.003
Urinary protein	−0.003	0.976
R^2^	0.365 (p < 0.001)	

eGFR: estimated glomerular filtration rate, PTH: parathyroid hormone, *β*: standardized correlation coefficient, R^2^: multiple coefficient of determination.

### Multivariate analyses to elucidate renal anemia parameters associated with serum f(HNA) in CKD patients

Renal anemia-related parameters, such as hemoglobin and ferritin, were significantly correlated with serum f(HNA) in the present patients (Table 2). To further identify renal anemia-related parameters independently associated with serum f(HNA), we performed additional multivariate analyses using the parameters ferritin, transferrin saturation (TSAT), use of erythropoiesis-stimulating agent (ESA), and iron supplementation including iron-based phosphate binders as explanatory variables. In addition to age, eGFR, and hemoglobin, the level of serum ferritin was significantly and independently associated with serum f(HNA) in all 4 models, whereas TSAT, use of ESA, and iron supplementation were not (Table 4). When CKD patients were subdivided into 4 groups according to the median values for hemoglobin (12.4 g/dL) and ferritin (100 ng/mL), f(HNA) in patients with higher hemoglobin and lower ferritin levels was significantly lower as compared to those with lower hemoglobin and higher ferritin levels (Supplemental Fig. 1).

**Table 4:** Multivariate analyses of renal anemia-related parameters associated with serum fraction of human non-mercaptalbumin [f(HNA)].

	Model 1		Model 2		Model 3		Model 4	
	*β*	*p*	*β*	*p*	*β*	*p*	*β*	*p*
Age	0.200	0.014	0.198	0.017	0.200	0.014	0.201	0.015
Gender	−0.043	0.633	−0.044	0.628	−0.029	0.749	−0.043	0.634
Body mass index	0.120	0.155	0.121	0.155	0.116	0.1703	0.121	0.156
eGFR	−0.238	0.009	−0.237	0.009	−0.310	0.003	−0.236	0.010
Hemoglobin	−0.346	<0.001	−0.343	<0.001	−0.371	<0.001	−0.343	<0.001
Ferritin	0.200	0.019	0.209	0.031	0.231	0.009	0.199	0.022
Transferrin saturation	—	—	−0.018	0.851	—	—	—	—
Use of ESA	—	—	—	—	0.147	0.167	—	—
Iron supplementation	—	—	—	—	—	—	−0.013	0.871
R^2^	0.356 (p < 0.001)		0.357 (p < 0.001)		0.369 (p < 0.001)		0.357 (p < 0.001)	

eGFR: estimated glomerular filtration rate, ESA: erythropoiesis-stimulating agent, *β*: standardized correlation coefficient, R^2^: multiple coefficient of determination

### Multivariate analyses to elucidate CKD-MBD parameters associated with serum f(HNA) in CKD patients

CKD-MBD related markers, such as phosphate, intact-PTH, and FGF-23, were significantly correlated with serum f(HNA) in the present CKD patients (Table 2). Therefore, to further identify CKD-MBD-related parameters independently associated with serum f(HNA), we performed additional multivariate analyses with the parameters corrected calcium, phosphate, intact-PTH, FGF-23, and 1,25-dihydroxyvitamin D (1,25(OH)_2_D) included as explanatory variables. Our results showed that intact-PTH was significantly and independently associated with serum f(HNA) (Model 1) (β = 0.218, p = 0.049) (R^2^ = 0.339, p < 0.001), whereasFGF-23 was not (Model 2) (β = 0.084, p = 0.476) (R^2^ = 0.317, p < 0.001). Notably, 1,25(OH)_2_D was significantly and inversely associated with f(HNA) in our CKD patients in analyses with both Model 1 and 2 (Table 5).

**Table 5:** Multivariate analyses of various CKD-MBD-related factors associated with serum fraction of human non-mercaptalbumin [f(HNA)].

	Model 1		Model 2	
	*β*	*p*	*β*	*p*
Age	0.310	<0.001	0.276	0.002
Gender	−0.038	0.673	0.001	0.993
eGFR	−0.285	0.009	−0.338	0.004
Corrected calcium	0.079	0.458	−0.015	0.895
Phosphate	0.060	0.566	0.023	0.848
Intact-PTH	0.218	0.049	—	—
Fibroblast growth factor-23	—	—	0.084	0.476
1,25(OH)_2_D	−0.178	0.040	−0.176	0.049
R^2^	0.339 (p < 0.001)		0.317 (p < 0.001)	

CKD-MBD; chronic kidney disease and mineral and bone disorder, eGFR: estimated glomerular filtration rate, and PTH: parathyroid hormone, 1,25(OH)_2_D: 1,25-dihydroxyvitamin D, *β*: standardized correlation coefficient, R^2^: multiple coefficient of determination.

## Discussion

In the present study, we investigated factors associated with serum levels of HNA (oxidized form of serum albumin) and HMA (reduced form of serum albumin) in pre-dialysis CKD patients using a newly established, highly sensitive assay based on an anion-exchange column packed with a hydrophilic polyvinyl alcohol gel, along with HPLC. Our findings clearly demonstrated that decreased renal function, represented by lower eGFR, was strongly associated with f(HNA) in pre-dialysis CKD patients. We also found that the serum level of f(HNA) was significantly higher in CKD patients with anemia as compared to those without. Multivariate analysis revealed that age, renal function, hemoglobin, and ferritin levels were significantly and independently associated with serum f(HNA) after adjustment for other confounders. These findings suggest the importance of determining renal function, hemoglobin and ferritin levels for assessment of the redox state of serum albumin in CKD patients. In addition, the present results demonstrate that intact-PTH and 1,25(OH)_2_D are significantly and independently associated with f(HNA), for the first time.

In a previous study, the proportion of HNA, measured with a method different from ours, was demonstrated to be significantly increased in pre-dialysis CKD patients, and serum creatinine levels were significantly and positively correlated with f(HNA)^13^. Our result of significant association of renal function (eGFR) and f(HNA) was consistent with that of the previous study. Differences between the previous study^13^ and the present study were summarized in Supplemental Table 1. Firstly, in the present study, data for several CKD-related parameters not included in the previous study^13^ have been added, including eGFR, urinary protein, sodium-chloride, TSAT, use of ESA or iron supplementation, intact-PTH, FGF-23, 1,25(OH)_2_D, plasma glucose, and hemoglobin A1c. Those parameters as well as others were evaluated in the present study in relation to the level of f(HNA). We consider that measurement of those markers in the present study is one of the advantages. Secondary, the results of multivariate analyses were different. We found that in addition to eGFR, hemoglobin, ferritin, intact-PTH, and 1,25(OH)_2_D were also significantly associated with f(HNA) in our CKD patients. In the previous study, multivariate analysis showed that hemoglobin was not significantly associated with f(HNA), while creatinine and uric acid each had a significant association^13^. In general, oxidative stress is increased with age. In our study, age was found to be a significant factor associated with f(HNA), in agreement with several other reports^18–20^, whereas the previous study did not find such a significant association^13^. This difference might have been due to the fact that the number of CKD patients in their study (n = 55) was fewer as compared to ours (n = 112). Another explanation for these different findings may be due to differences in the analytical methods used for f(HNA). Novel findings in our study include the results of multivariate analysis showing that hemoglobin, ferritin, intact-PTH, and 1,25(OH)_2_D were significantly associated with f(HNA) in CKD patients. Finally, the measurement method of f(HNA) was different. With our method, the measurement time is far shorter, and the accuracy is very high with the CV values of 0.3%^12^.

In our previous study, f(HNA) in healthy subjects was 25.1 ± 3.0%^9^, while that in the present pre-dialysis CKD patients was 30.0 ± 6.1%, higher than in those healthy subjects. In another study, though the method used for measurement was different, f(HNA) in patients undergoing hemodialysis was 41.5%^21^, higher than the value for pre-dialysis CKD patients in the present study. In addition, eGFR was significantly and inversely associated with f(HNA) in our study. These findings suggest that decreased renal function is associated with oxidization of serum albumin, shown by f(HNA) level.

In previous studies that used conventional measurement methods, serum f(HNA) was found to be increased with aging in healthy subjects^18–20^. With our novel assay method for HNA and HMA, age was also significantly and positively correlated with f(HNA) in healthy subjects^9,12^. Although the age of the healthy subjects (63.1 ± 9.9 years) in those previous studies was similar to that of the present patients (63.6 ± 14.0 years), f(HNA) in the present study was 30.0 ± 6.1%, higher as compared to our previous healthy subjects. Furthermore, strong and significant correlations were observed between f(HNA) and eGFR, and between f(HNA) and age in this study, suggesting a close correlation of f(HNA) with oxidative stress, renal function, and age. Also, our multiple regression analysis results demonstrated for the first time that age and renal function are significantly and independently associated with f(HNA).

In patients with ESRD undergoing hemodialysis, f(HNA) has been reported to be decreased from approximately 40% before to 30% after a dialysis session^22^. That study also noted that such a rapid alteration of redox state of serum albumin before and after dialysis resembled the alteration of serum urea nitrogen levels seen with hemodialysis treatment. Furthermore, a significant reduction in oxidized albumin ratio was reportedly observed after administration of AST-120, a carbonaceous adsorbent drug, in 5/6 nephrectomized CKD rats^23^. In the present study, blood urea nitrogen was significantly and highly correlated with serum f(HNA). Among uremic toxins, indoxyl sulfate is known to induce oxidative stress via promotion of free radical production^24^. In a previous study of CKD rats, exogenous administration of indoxyl sulfate increased the ratio of oxidized albumin^23^. Furthermore, AST-120 was found to decrease serum indoxyl sulfate levels in CKD patients receiving dialysis^25^ as well as pre-dialysis patients with CKD^26^. Together, these findings suggest that administration of AST-120 may reduce f(HNA) in CKD patients. Nevertheless, additional longitudinal studies to confirm the effects of AST-120 on the redox state of serum album are considered to be clinically necessary.

In pre-dialysis CKD patients, the present results showed that f(HNA) is significantly and independently associated with hemoglobin and ferritin. That positive and significant association between serum ferritin, a marker of iron status of CKD^27^, and f(HNA) suggests that increased iron storage impairs the redox state of serum albumin. Previously, free iron was shown to be a powerful source of hydroxyl radicals through the Fenton reaction^28^, while iron loading alters anti-oxidant systems^29^. Furthermore, emerging evidence supporting the role of iron overload in cardiovascular complications has been presented^30,31^, which is thought to be due to endothelial dysfunction induced by ROS increase^32^. Indeed, serum f(HNA) in hemodialysis patients receiving intravenous iron administration was significantly higher (41.7 ± 6.3%) as compared to those without that administration (36.0 ± 6.0%)^21^. Nevertheless, we did not find a significant association of oral iron supplementation with f(HNA) in our study. A recent investigation conducted to compare the effects of oral and parenteral iron in a CKD population was terminated early, because the group receiving parenteral iron supplementation experienced a 2.51-fold higher incidence of CVD events and a 2-fold greater incidence of hospitalization for heart failure as compared with the oral iron-treated group^33^. Those findings indicate that oral iron supplementation for maintaining an adequate ferritin level may not be so harmful, at least as compared to parenteral iron supplementation. In contrast, TSAT is used as an index for responsiveness to ESA rather than absolute iron deficiency, because TSAT results show a large circadian variation and are easily affected by factors other than iron status, such as inflammation and nutritional status^34^. Thus, we consider that TSAT is not associated with f(HNA), even though ferritin showed a significant association with f(HNA) in the present study. Additional studies that examine a larger number of CKD patients are needed in order to investigate the effects of ESA and oral or parenteral iron supplementation on serum albumin redox status.

As for factors related to CKD-MBD, both PTH^34,35^ and FGF-23^36,37^ have been shown to mediate oxidative stress. Thus, investigations of the association between CKD-MBD related markers and f(HNA) are important for treatment of CKD patients. In the present CKD patients, both simple and multiple regression analyses revealed that intact-PTH was associated significantly and positively with serum f(HNA). In contrast, multiple regression analysis findings showed that FGF-23 did not have a significant association. Intact-PTH was shown to be significantly correlated with inflammatory biomarkers, which are known to induce oxidative stress^35^. In addition, administration of paricalcitol, a selective vitamin D receptor activator, for 12 weeks reduced the levels of oxidative stress markers, including malondialdehyde, nitrites, and carbonyl groups, as well as intact-PTH levels in patients receiving hemodialysis^38^. Notably, serum 1,25(OH)_2_D, an active form of vitamin D, had a significantly negative association with f(HNA) in our multiple regression analysis results. Our new findings suggest that among CKD-MBD related factors, PTH and 1,25(OH)_2_D have effects on the redox state of serum albumin. Based on these results, treatment of CKD-MBD using vitamin D receptor activators, which reduce PTH and increase 1,25(OH)_2_D, may have an effect on amelioration of the redox state of serum albumin in affected patients. Nevertheless, a longitudinal study is necessary.

The present study has some limitations. First, the number of patients examined was relatively small, mainly because the subjects were enrolled from a single institution. Also, patients with liver dysfunction, reported to have effects on the redox state of serum albumin^8,19,39^, were excluded, because we intended to examine f(HNA) in CKD patients. Furthermore, this study had a cross-sectional design and the findings cannot be used demonstrate causality of the factors, i.e., we could not conclude that control of uremic toxins, such as using AST-120, control of renal anemia, such as with use of ESA, or decreased ferritin leads to decreased serum f(HNA). Additional studies that explore whether serum f(HNA) can be reduced through strict control of hemoglobin levels in CKD patients are necessary, particularly regarding the link between improvement of anemia and decrease in serum f(HNA), which could be confirmed in experimental animal models. Other *in vivo* studies may also be necessary to determine the potential effects of some uremic toxins and ferritin on f(HNA). Finally, ankle brachial pressure index (ABI) and (PWV) pulse wave velocity have been shown to be strong predictors of cardiovascular events, not only in the general population^40^, but also in CKD patients^41^. An additional longitudinal study is needed to investigate the association between f(HNA) and ABI/PWV.

In conclusion, this is the first study to show that serum f(HNA) is significantly associated with decreased renal function and renal anemia levels in CKD patients, independent of age. Our results also demonstrated that intact-PTH and 1,25(OH)_2_D are significant factors associated with serum albumin redox state. Together, the present findings suggest the importance of management of hemoglobin and ferritin levels, as well as appropriate control of CKD-MBD factors to provide a better redox state of serum albumin in CKD patients.

## Methods

### Ethics statement

The study protocol was approved by the Ethics Committee of Osaka City University Graduate School of Medicine (approval #603366). All study participants provided written informed consent for both collection of blood and urine samples, and examination of clinical records relevant to the present study. The research was conducted in accordance with the Declaration of Helsinki.

### Patients

CKD was defined according to criteria proposed by the Kidney Disease: Improving Global Outcomes Organization^42^. A total of 112 CKD patients with eGFR < 60 mL/min/1.73 m^2^ and regularly followed by nephrologists at Osaka City University Hospital were enrolled between March and April 2016. The etiologies of these patients included hypertensive nephrosclerosis (n = 21), IgA nephropathy (n = 16), membranous nephropathy (n = 16), diabetic nephropathy (n = 15), anti-neutrophil cytoplasmic antibody (MPO-ANCA)-associated glomerulonephritis (n = 7), autosomal dominant polycystic kidney disease (n = 7), tubulointerstitial nephritis (n = 5), minimal change nephrotic syndrome (n = 3), membranoproliferative glomerulonephritis (n = 3), purpura nephritis (n = 3), focal and segmental glomerulosclerosis (n = 2), lupus nephritis (n = 2), and unknown cause (n = 12). Since liver dysfunction has been reported to have an effect on the redox state of serum albumin^8,19,39^, patients with hepatitis C (n = 6), hepatitis B (n = 2), alcoholic liver cirrhosis (n = 2), fatty liver (n = 8), drug-induced liver injury (n = 4), and liver cancer (n = 4) were excluded from the present analysis (Supplemental Fig. 2).

### Blood sampling

Spot blood and urine samples were collected from all subjects in the morning after overnight fasting. The urine samples were kept on ice for 1 hour and then centrifuged at 630 × g for 10 minutes, as previously described^43^. All laboratory measurements were performed using routine assays with automated methods^43^. Estimated glomerular filtration rate (eGFR) was calculated using the new Japanese coefficient for the abbreviated Modification of Diet in Renal Disease Study equation, including a correction factor of 0.739 for females^44^. Serum calcium was corrected based on serum albumin and determined as corrected calcium, as previously described^45^. Serum intact- PTH was measured using a second-generation Elecsys PTH IRMA assay (Roche Diagnostics, Mannheim, Germany), as previously reported^45^. Serum full-length fibroblast growth factor-23 (FGF-23) was measured using a CL-JACK System (Kyowa Medex Co. Ltd., Tokyo, Japan), a fully automated random access chemiluminescence immuno-analyzer, as previously reported^45^. Serum 1,25(OH)_2_D was measured with a 1,25(OH)_2_D RIA kit (Immunodiagnostic Systems Limited, Boldon, England), as previously described^45^.

### Measurements of human non-mercaptalbumin (HNA) and mercaptalbumin (HMA)

Measurements of HNA were performed using freshly frozen samples kept at −80 °C until the time of the assay utilizing the recently established assay that employs anion-exchange HPLC^9,12^. A LabSolutions system (Shimazu Co., Ltd., Kyoto, Japan), consisting of a degasser (DGU20A3R), 2 pumps (LC-20AT), an auto-sampler (SIL30AC), a thermostatic oven (CTO-20AC), a fluorescence detector (RF-20Axs), and a system controller (CBM-20A), was used for the present study^9,12^.

The gel and column for analysis of HNA and HMA were produced as follows. A polyvinyl alcohol cross-linked gel (9 µm in diameter) (Asahipak GS-520, Asahi Kasei Co., Ltd., Tokyo, Japan) was dried in a vacuum for more than 16 hours, then suspended in 10 ml of dimethyl sulfoxide (Tokyo Chemical Industry Co., Ltd., Tokyo, Japan) per 1 g of dried gel. Next, 20 mmol of epichlorohydrin for each 1 g of dried gel was added to suspend the gel and the reaction was allowed to proceed for 20 hours at 30 °C. The activated gel was subsequently filtrated and reacted with a 10% aqueous solution of diethyl amine (Wako Pure Chemical Ind., Co., Ltd, Osaka, Japan) for 20 hours. The synthesized anion-exchange gel was then packed into a stainless column (50 × 7.6 mm I.D.).

HPLC analysis conditions were as follows. The solution of 25 mM phosphoric acid buffer containing 60 mM sodium sulfate (pH 6.0) was employed for the first eluent (eluent A) to absorb and separate the albumin fraction. A high concentration magnesium chloride solution was used for eluting HMA and HNA from the column as an eluate (eluent B). The flow rate was set at 1 mL/min after equilibrating the column for 4.5 min with eluent A. The linear gradient time program from eluent A (100%) to eluent B (100%) lasted for 7.5 min and started at the time of sample injection. The total measurement time was 12 min per sample, including both column equilibration and the analysis time. 3 mL of serum was used for analysis. Analysis temperature was 40 °C. The influence of substances interfering with this measurement method was examined using the actual HPLC chromatograms and no abnormal chromatograms with effects on the measurement results were found.

### Statistical analyses

Statistical analyses were performed using Graphpad Prism version 6.0 for Windows (Graphpad Software, San Diego, CA) and JMP software version 10 (SAS Institute Inc., Cary, NC). Values are expressed as the mean ± SD. Comparisons of f(HNA) between CKD patients with and without renal anemia were made using an unpaired Student’s t-test or Mann-Whitney U test for continuous variables, or a chi-square test for categorical variables. One-way ANOVA was used to assess difference in mean values between groups. Correlations between human f(HNA) and clinical data were examined by Spearman’s analysis. Independent associations between clinical variables and f(HNA) in the present CKD patients were assessed by multiple regression analyses, with dummy variables of 0 and 1 included for users and non-users of ESA, respectively, and iron supplementation (yes, no), respectively. BMI, renal function, AST and uric acid levels were found to be significantly correlated with f(HNA) in healthy Japanese subjects^9^, while hemoglobin and serum uric acid were shown to be significantly correlated with f(HNA) in CKD patients^13^. Furthermore, chronic metabolic acidosis has been shown to decrease albumin synthesis^46^. Since intact-PTH was found to be significantly correlated with inflammatory biomarkers, which induce oxidative stress^35^, that may also have an effect on the redox state of serum albumin. Additionally BMI, renal function, AST, uric acid, sodium-chloride, and intact-PTH may also have effects on the redox state of serum albumin, thus those parameters were added as explanatory variables for multivariate analysis (Table 3). P values < 0.05 were considered to indicate statistical significance. The datasets generated and analyzed for the current study are available from the corresponding author upon reasonable request.

## Electronic supplementary material

Supplemental information
