# Supplementary material for: Non-mercaptalbumin, Oxidized Form of Serum Albumin, Significantly Associated with Renal Function and Anemia in Chronic Kidney Disease Patients
Source: Sci Rep. 2018 Nov 14;8:16796. doi: 10.1038/s41598-018-35177-x (PMC6235854; doi:10.1038/s41598-018-35177-x)
Supplement: Supplementary file 1 — Supplemental information [file 41598_2018_35177_MOESM1_ESM.pdf]

Non-mercaptalbumin, Oxidized Form of Serum Albumin, Significantly Associated with Renal Function and Anemia in Chronic Kidney Disease Patients

Shinya Nakatani[1†], Keiko Yasukawa[2], Eiji Ishimura[3*†], Ayumi Nakatani[1], Norikazu Toi[1], Hideki Uedono[1], Akihiro Tsuda[1], Shinsuke Yamada[1], Hitoshi Ikeda[2], Katsuhito Mori[3], Masanori Emoto[1],Yutaka Yatomi[2], and Masaaki Inaba[1, 3]

[1]Department of Metabolism, Endocrinology, and Molecular Medicine, Osaka City University Graduate School of Medicine, Osaka, Japan

[2]Department of Clinical Laboratory, The University of Tokyo Hospital, Tokyo, Japan

[3]Department of Nephrology, Osaka City University Graduate School of Medicine, Osaka, Japan

[*] *Corresponding author and address for reprint requests*:

Eiji Ishimura, MD, PhD, FASN, FACP

Department of Nephrology, Osaka City University Graduate School of Medicine

1-4-3, Asahi-machi, Abeno-ku, Osaka 545-8585, Japan

Tel: +81-6-6645-3806, Fax: +81-6-6645-3808, Email: ish@med.osaka-cu.ac.jp

[†] SN and EI contributed equally to this work.

***Word count*:** Title, 18 words; Abstract, 200 words; Main text 2824 words

Tables and Figures: 5 tables, 1 figure, 1supplemental table and 2supplemental figure

Supplemental Table 1. Differences between the previous study and ours.

Study	Terawaki H, et al. (Kidney Int 2004)	Nakatani S, et al.
Design	Cross-sectional study	Cross-sectional study
Number of CKD patients	55	**112**
Measurement of fraction HNA	Conventional method (Hayashi T, et al. Pathophysiology 2000)	**Novel method** (Yasukawa K, et al. Ann Clin Biochem 2018)
Measurement time	Time consuming	**Shorter**
Accuracy	CV value not available	**CV value less than 0.3%**
Laboratory measurements		
Creatinine (mg/dL)	Yes	Yes
**Creatinine clearance (mL/min) (evaluated by Cockcroft-Gault formula)**	**Yes**	No
**eGFR (mL/min/1.73 m^2)**	No	**Yes**
Blood urea nitrogen (mg/dL)	Yes	Yes
**Urinary protein (g/gCr)**	No	**Yes**
Aspartate transaminase (IU/L)	No	Yes
Alanine transaminase (IU/L)	Yes	Yes
Total protein (g/dL)	Yes	Yes
Albumin (g/dL)	Yes	Yes
C-reactive protein (mg/dL)	Yes	Yes
Uric acid (mg/dL)	Yes	Yes
Sodium (mEq/L)	Yes	Yes
Potassium (mEq/L)	Yes	Yes
Chloride (mEq/L)	Yes	Yes
**Sodium-chloride (mEq/L)**	No	**Yes**
**Salt excretion (g/day)**	**Yes**	No
**Estimated protein intake (g/kgBW/day)**	**Yes**	No
Diabetes-related markers		
**Plasma glucose (mg/dL)**	No	**Yes**
**Hemoglobin A1c (%)**	No	**Yes**
Anemia-related markers		
Hemoglobin (g/dL)	Yes	Yes
Total iron (μg)	Yes	Yes
**Ferritin (ng/mL)**	No	**Yes**
**Transferrin saturation (%)**	No	**Yes**
**ESA**	No	**Yes**
**Iron supplementation**	No	**Yes**
CKD-MBD-related markers		
Corrected calcium (mg/dL)	Yes	Yes
Phosphate (mg/dL)	Yes	Yes
**Intact-PTH (pg/mL)**	No	**Yes**
**FGF-23 (pg/mL)**	No	**Yes**
**1,25$(OH)_2$D (pg/mL)**	No	**Yes**
Results of multivariate analysis (factors associated with f(HNA))		

**Age**	Not significant	**Significantly associated**
**eGFR**	Not measured	**Significantly associated**
**Creatinine**	**Significantly associated**	Not included<br>(eGFR included)
**Hemoglobin**	Negative	**Significantly associated**
**Intact-PTH**	Not measured	**Significantly associated**
**Uric aid**	**Significantly associated**	Negative

GFR: estimated glomerular filtration rate, ESA: erythropoiesis-stimulating agent, FGF23: fibroblast growth factor-23, PTH: parathyroid hormone, 1,25$(OH)_2$D: 1,25-dihydroxyvitamin D

Supplemental Figure 1

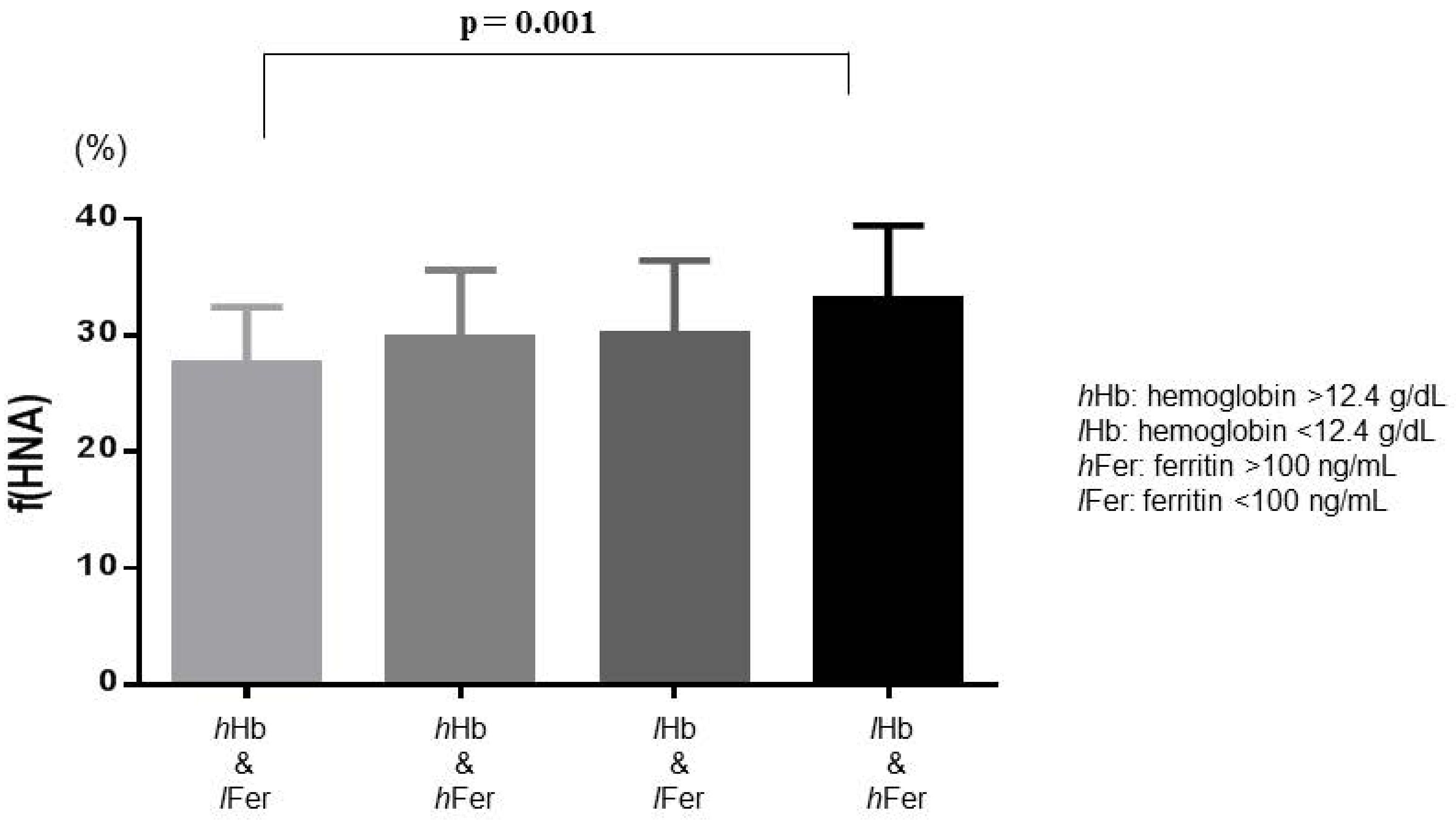

Supplemental Figure 2

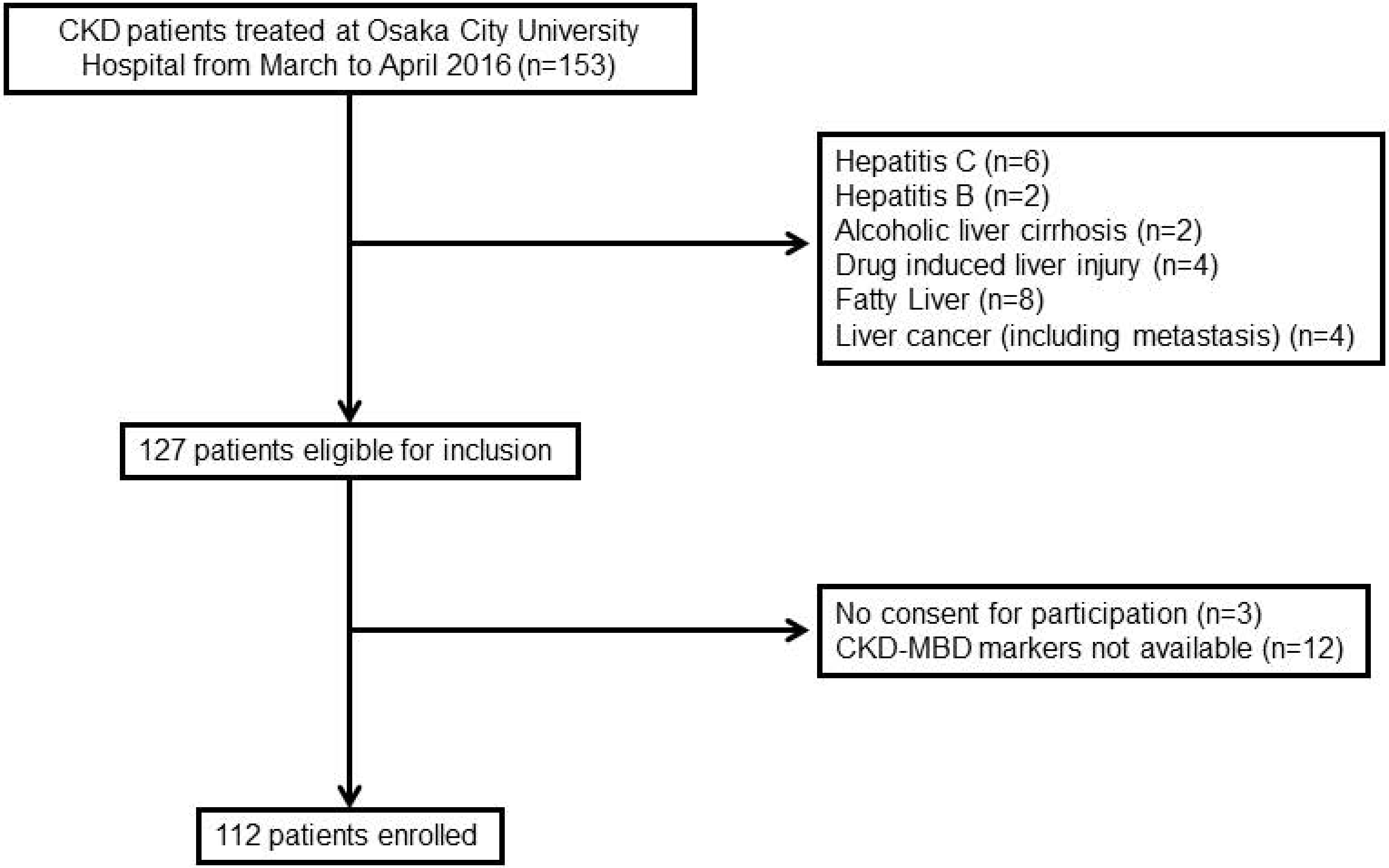

Figure legends

Supplemental Figure 1. Comparisons of fraction of human non-mercaptalbumin [f(HNA)] with hemoglobin and ferritin levels in CKD patients. f(HNA) in CKD patients with lower hemoglobin and higher ferritin was significantly elevated as compared to that in those with higher hemoglobin and lower ferritin levels.

Supplemental Figure 2. Patient enrollment. This was a cross-sectional single center study of 112 CKD patients with eGFR <60 mL/min/1.73 m^2. Patients with liver dysfunction, such as hepatitis C (n=6), hepatitis B (n=2), alcoholic liver cirrhosis (n=2), fatty liver (n=8), drug-induced liver injury (n=4), and liver cancer (n=4), were excluded.
